# Commentary on: #PRS: A Study of Plastic Surgery Trends With the Rise of Instagram

**DOI:** 10.1093/asjof/ojad017

**Published:** 2023-02-16

**Authors:** Daniel J Gould

In this article,^1^ the authors look at the inflection points in the search indexes for different type of procedures over time (Video). They identify interesting trends in search associated with procedures, and potential links between the social media platforms and public interest in procedures. What may also be reflected is changes in the social media platforms themselves and the types of content they allow to be seen and promote.

In the modern world of social media, as algorithms change it is the duty and the responsibility of plastic surgeons to continue to improve transparency around procedures, including not just showing the potential for surgical outcomes but also the potential complications and long-term results. Safety and the process of surgery needs to be highlighted.

## Supplementary Material

ojad017_Supplementary_Data
