# Supplementary material for: Commentary on: #PRS: A Study of Plastic Surgery Trends With the Rise of Instagram
Source: Aesthet Surg J Open Forum. 2023 Feb 16;5:ojad017. doi: 10.1093/asjof/ojad017 (PMC10120159; doi:10.1093/asjof/ojad017)
Supplement: ojad017_Supplementary_Data [file ojad017_Supplementary_Data.zip › 23-0015_video transcription.docx]

**Transcription**

In this recent article published in ASJ, the authors looked at the rise of plastic surgery trends and how they coincide with Instagram as a social media platform. So it's an interesting article where essentially the authors looked at an inflection point in 2012, which was about the time when Instagram was acquired by Facebook and really became a dominant force in social media.

The question that the authors asked was, if we look at Google Trends, which is a way to map the public's interest in different types of procedures, how do those change from 2004 to 2012 compared to 2012 to 2020? In this study, what they identified is that there were several big search topics and questions that did increase over time after that inflection point.

What's interesting is the terms that they looked at included other subgroups of terms. So, for instance, they looked at the question for rhinoplasty. They also looked at nose job and other words that were associated in order to fully capture a picture of the public's interest in this type of procedure. The authors did show that there were positive trends over time, and what the authors state in the discussion is that they think that Instagram had some influence in 2012 in improving the public's interest in these types of procedures.

Now, what's more interesting in the article is the type of questions that they don't answer and the things that they didn't look at. So, for instance, breast augmentation had a downward inflection point. Buccal fat was a downward inflection point, abdominoplasty was relatively stable. And this is in contrast to brachioplasty, which went up over time.

You would think that as patients are interested in weight loss procedures or as their interest gains, that they'd be looking at thighplasty, brachioplasty and tummy tuck, where thighplasty interests went up, brachioplasty interest went up, abdominoplasty interest stayed relatively stable over time. So my question is, why is it that those types of procedures did not increase in the inflection point with Instagram.

Breast augmentation and abdominoplasty were relatively stable. Also, buccal fat seemed to go down. I think if the authors expanded the length of time and they looked at the last year, the Google Trends search for buccal fat is probably up because of recent interest that was really popularized even within the last few months.

The authors do state that, you know, public's interest can be it can be influenced by what's going on in the news, what's going on in pop culture, and that that's probably responsible for some of the findings and some of the rapid inflection points that they see over time. My secondary question is, are the platforms and what they allow us to show affecting the trends?

So maybe breast augmentation stays relatively stable because of Instagram's shadow banning of those types of before and after photos. Maybe there's some kind of censorship that's going on that's preventing the reach of certain types of before and after photography and interest in these types of procedures. The above the neck types of procedures were widely upward trending, with the exception of buccal fat. Virtually every other procedure that's above the neck showed increase in interest, and it was much more of an inflection, much more of a linear improvement. Statistically, I think the paper's good, but I think they could strengthen the statistics of the paper by looking at linear regression models and doing a little bit more of a deep dive in the bio statistics, not simply looking at whether or not it's an upward inflection or whether or not that's significant.

Maybe some linear regression analysis would really look at the deeper kind of questions that we have about each of these types of increases in interest over time. Regardless, my other question is, if 2012 is the inflection point and it's a linear inflection, how do you know it's 2012? Do we see trends that go down before that or stable before that and then take off? How do I identify that 2012 point as the inflection point? And Instagram has increased in popularity. So as Instagram's increased almost exponentially between 2012 and 2018, were there likewise exponential increases in these types of interest in procedures because you'd think that the two would be directly correlated.

Regardless, it's a great study because it does show that over time the type of interest that we see which has gone up, which we all see in our practices, can be traced with Google Trends, and that's been proved by other authors before.

But this further kind of reinstates and further reinforces the concept that research can be done to look at the public's interest. And this can be matched with actual changes of what we see in practice. Also, there is some interest in looking at that time frame 2004, and 2022 actually. I think there's a nice way to look at the the way that the interest increases over time and to say, hey, how did these different social media platforms affect interest at each point?

I'd really like to do a study where we look even closer at the adaptation of for instance, video to Instagram, the adaptation of new services that the different social media platforms allow us to utilize, co-branding, other types of things that can change dramatically what patients can see and what they can hear with each of the posts. If they did a study where they looked at the adaptation and the addition of transcription of different videos, that would also be very interesting.

So, this it's just some food for thought. I think that the authors have really taken a nice dive to look at the surface level, how Google Trends maps with the adaptation and addition of Instagram to social media and the growth of the population's interest in plastic surgery procedures over time. But we need to go a little further, and I think that we all are going to be learning a lot more over the next 10 to 15 years about how we can specifically utilize these social media platforms in order to increase the visibility of the types of procedures that we have and to really inform the public not just to allow them to search and identify things they're interested in, but to help guide them to things that are safe, outcomes that are good, practitioners that are skilled and ultimately safe results that will last them over time and a happy outcome with their plastic surgical procedures.

So, kudos to the authors and big congratulations to the Aesthetic Surgery Journal for publishing this study for taking time to really look deeply at how social media is affecting our practices. I look forward to many more of these studies from these authors to come.
